# Supporting workers with mental health problems at work: challenges and avenues

**DOI:** 10.5271/sjweh.4044

**Published:** 2022-06-30

**Authors:** Iris Arends, Sander KR van Zon, Ute Bültmann

**Affiliations:** 1 Department of Health Sciences, Community and Occupational Medicine, University of Groningen, University Medical Center Groningen, Groningen, The Netherlands; 2 Arbo Unie, Utrecht, The Netherlands

Mental health problems in the workforce present a major public and occupational health challenge and come with significant costs for the individuals, families, employers and society at large. It has been estimated that, globally, the 12-month prevalence of common mental health problems – such as depressive disorders, anxiety disorders, and stress-related disorders – is on average 17.6%, with often serious implications for employment, productivity, and wages (2, 3). The recent OECD report “Fitter Minds, Fitter Jobs” showed that in 2018, across OECD countries, people with mental health problems have 20% lower employment rates, are almost three times more likely to be unemployed, and almost one and a half times more likely to receive disability benefits as those without these problems (2). These key figures barely differ from those presented in 2013 (2).

During the past decades, research on the highly complex phenomenon of (return to) work participation of people with common mental health problems has come a long way: many barriers and facilitators to (return to) working have been identified and interventions have been developed and tested for people with common mental health problems to participate in work (eg, 4, 5). To illustrate, facilitating factors concern, for example, an individual’s active coping style (keep a daily rhythm, exercise, stay in contact with work), high self-efficacy, and a supportive family context and social network (5-8); while the severity of mental health problems or the existence of other health problems are known barriers (5). A safe organizational climate (such as openness about mental health) and good psychosocial working conditions, including support from supervisors and colleagues, having decision authority, and no high workload, have been identified as facilitating workplace factors (5, 7, 8). Also, health and social systems may act as a barrier or facilitator with, eg, waiting lists for mental health treatment or the availability of integrative mental health and occupational rehabilitation/employment services (7, 9). It comes as no surprise that Corbière et al (10) identified 11 different stakeholder groups from the work, health and insurance systems and close to 200 relevant stakeholder actions in the return-to-work process of workers with common mental health problems.

Despite extensive progress and a large body of evidence on factors to facilitate the (return to) work participation of workers with common mental health problems (for systematic reviews and meta-analyses, covering more than two decades of research, see, eg, 5, 9, 11), we must acknowledge that meta-analyses of intervention studies to date only have shown small effect sizes for sick leave reduction (4, 12–14) and no substantial effects for improved return-to-work (13) or being at work (14) rates. So, how to move the research field forward? Although people with common mental health problems have lower employment rates, the majority (60% on average across OECD countries) is working (OECD 2021), but knowledge about maintaining and improving at-work participation among this group is lacking. We see a great need for a focus shift towards a deeper understanding of at-work participation of people with common mental health problems. In the following, we focus on two challenges and avenues to move forward: (i) measuring at-work outcomes and (ii) examining the complex, interdependent relationship between common mental health problems and at-work participation with more intense, longitudinal real-time designs and a life course lens.

**Challenges and avenues to support people with common mental health problems at work**

***Challenge 1: Measurement of at-work outcomes***

The first challenge concerns the measurement of how people with common mental health problems participate or function at work and what their needs are to enter and stay at work. To better support workers with common mental health problems at work, it is critical to further deepen our understanding of the strategies, work accommodation needs and functioning of these workers. To illustrate, in a qualitative study among workers suffering from common mental health problems, Danielsson et al (15) explored “strategies to keep working”. The authors showed that workers’ strategies differed depending on the illness phase; ie, more reactive strategies to avoid strain were used in early phases and more reflective, solution-focused strategies were used in later phases. This knowledge on phase-specific work strategies may be used to better inform and tailor supportive interventions and work accommodation to help workers to maintain working. De Groot et al (16) recently provided first insights about how young adults with a history of mental health problems function at work. It was shown that young adults with both persistent high and elevated levels of mental health problems during childhood and adolescence, compared with those with low-level mental health problems, experience difficulties in meeting their work demands for more than one day a week given a full-time work week at age 29. Moreover, Arends et al (17) showed that many workers who returned to work after being absent with common mental health problems still experience impaired work functioning for up to 12 months. This study also demonstrated that workers recover at a different pace and at a different level in terms of mental health and work functioning.

These findings highlight the importance of focusing on at-work strategies and functioning to support workers with common mental health problems as we need to capture early signs of maladaptive strategies or reduced functioning that may inform work accommodations to prevent a further decline in functioning or even more severe consequences as sick leave or work disability. Accommodating work for workers with (common) mental health problems may be especially challenging, as opposed to other health conditions, given the strong stigma attached to mental ill-health (18, 19). As discussed by LaMontagne et al (20), an integrated intervention approach to workplace mental health, combining knowledge from various disciplines (eg, occupational medicine, psychiatry, public health, -positive- psychology) and focusing on both protecting and promoting mental health as well as addressing mental health problems is essential (20).

To assess and monitor the abilities to accomplish the work role, it is vital to consider at-work outcomes, such as health-related work functioning, work limitations, work instability, and work capabilities (21–24). Ideally, such outcomes – existing or to be developed – are at the intersection of a persons’ health and work performance, reflect the ability and/or need of a person to meet the work demands given the available personal and/or environmental resources, and provide information for the content and timing of work accommodations. We strongly encourage future research to (further) rigorously test the measurement properties of existing and to be developed at-work measures, in particular the responsiveness to change, within the population of workers with common mental health problems.

***Challenge 2: Examination of the complex, interdependent relationship between common mental health problems and at-work participation: novel designs and a life course lens***

The second challenge concerns the need to better understand the complex, interdependent relationship between common mental health problems and at-work participation. To provide adequate and timely support for workers with common mental health problems at work, it is critical to further unravel the underlying mechanisms and (environmental) conditions of this complex, dynamic relationship, as different support policies and programs need to be in place at different time periods to address either common mental health problems or at-work participation. We would like to encourage future longitudinal studies to not shy away from complexity but to use approaches that capture the dynamics of both common mental health problems and at-work participation by, eg, repeatedly and more intensively assessing both concepts over time.

Not new, but to be considered in occupational health research and practice, may be the use of intensive, longitudinal real-time designs, as recently applied in single-subject time-series studies in psychiatry, addressing psychopathology as a complex system (eg, 25, 26). For example, to detect personalized early warning signals preceding the occurrence of a major depressive symptom transition, Wichers et al (26) conducted six single-subject time-series studies over a 3–6-month period, prospectively collecting frequent observations of momentary affective states (reported up to three times a day) during a time period when participants were at increased risk of a depressive symptom transition (reported weekly). The results showed (and replicated) the presence of rising early warning signals a month before the symptom transition occurred. To improve personalized support of workers with common mental health problems at work, this type of information is highly needed.

What makes the relationship between common mental health problems and at-work participation even more complex is the fact that a person’s mental health does not start when work begins; ie, what happens before a person enters the workforce affects both the health resources a person brings to work and the work opportunities (27). As most research so far measured common mental health problems during working adults’ life, and not across the life course, knowledge on the impact of early life mental health experiences on at-work participation in later life is almost absent. Again, the findings of de Groot et al (16) highlight the importance of adopting a life course perspective by considering the concept of ‘accumulation of health risk or health advantages’ when connecting early life mental health experiences with work functioning. A life course perspective may also help advance future studies on the dynamics between different life domains (7), eg, the interplay between work and private life, as it recognizes an individual’s life course as a multi-level developmental process shaped by the social context.

A focus shift towards supporting workers with common mental health problems at work also requires all key stakeholders in the healthcare system, the legal/administrative system, the work system and the personal and family system to work together – which may be a challenge in itself. However, in view of more inclusive workplaces and labor markets, we need to take the next steps to enable, maintain and improve at-work participation of workers with common mental health problems.
